# TakoTsubo Cardiomyopathy A Short Review

**DOI:** 10.2174/1573403X11309030003

**Published:** 2013-08

**Authors:** Shahbaz Roshanzamir, Refai Showkathali

**Affiliations:** Department of Cardiology, The Essex Cardiothoracic Centre, United Kingdom

**Keywords:** Apical ballooning syndrome, broken heart syndrome, catecholamine, oxidative stress, right ventricular dysfunction, Stress cardiomyopathy, takotsubo cardiomyopathy.

## Abstract

Takotsubo cardiomyopathy (TCM), otherwise cardiomyopathy,apical ballooning syndrome or broken heart syndrome is a reversible cardiomyopathy, predominantly occurs in post-menopausal women and commonly due to emotional or physical stress. Typically, patients present with chest pain and ST elevation or T wave inversion on their electrocardiogram mimicking acute coronary syndrome, but with normal or non-flow limiting coronary artery disease. Acute dyspnoea, hypotension and even cardiogenic shock may be the presenting feature of this condition. The wall motion abnormalities typically involve akinesia of the apex of the left ventricle with hyperkinesia of the base of the heart. Atypical forms of TCM have also recently been described. An urgent left ventriculogram or echocardiogram is the key investigation to identify this syndrome. Characteristically, there is only a limited release of cardiac enzymes disproportionate to the extent of regional wall motion abnormality. Transient right ventricular dysfunction may occur and is associated with more complications, longer hospitalisation and worse left ventricular systolic dysfunction. Recently, cardiac MRI has been increasingly used to diagnose this condition and to differentiate from acute coronary syndrome in those who have abnormal coronary arteries. Treatment is often supportive, however beta-blocker and angiotensin-converting enzyme inhibitor or angiotensin II receptor blocking agent are being used in routine clinical practice. The syndrome is usually spontaneously reversible and cardiovascular function returns to normal after a few weeks. This review article will elaborate on the pathophysiology, clinical features including the variant forms, latest diagnostic tools, management and prognosis of this condition.

## INTRODUCTION

**Fig. (1):**
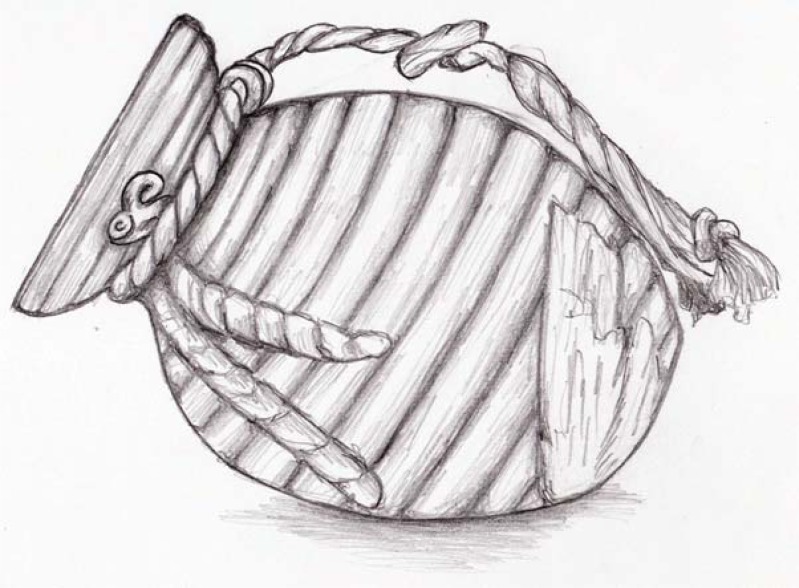
example of ‘takotsubo’, or octopus fishing pot.

‘Takotsubo’ cardiomyopathy (TCM) is a relatively new anomaly first described in Japan by Sato in 1990 [1]. His colleague Dote in 1991 named it Takotsubo because the shape of the left ventricle resembles a Japanese octopus trap, with a round bottom and narrow neck (Fig **1**) [2].

Takotsubo Cardiomyopathy is also referred to as stress cardiomyopathy, ‘transient apical ballooning’ or ‘broken heart’ syndrome. The cardinal feature of TCM is transient and reversible left ventricular (LV) dysfunction triggered by severe emotional or physical stress, in the presence of unobstructed coronary arteries. The exact stressors and pathogenesis remains unclear. Variant forms of left ventricular dysfunction have been reported, including wall-motion abnormalities, such as mid-ventricular ballooning with sparing of the basal and apical segments, or inverted Takotsubo. Right ventricular involvement is also reported in TCM [3]. Ventricular dysfunction is transient with resolution generally achieved within days or weeks. The disease is associated with excessive sympathetic stimulation, microvascular dysfunction, coronary artery vasospasm, and abnormal myocardial tissue metabolism [4]. An excessive release of catecholamines also seems to have a pivotal role in the development of TCM. This review summarizes published data on TCM, focusing primarily on the most likely causes of this relatively young but increasingly recognised and reported cardiac entity.

## PREVALENCE

To date, there have been little more than 1200 Pubmed published reports on TCM. The prevalence among patients with symptoms suggestive of acute coronary syndrome is 1.0–2.5%, with almost 90% of cases being in post-menopausal women [4, 5]. Overall, in ST elevation myocardial infarction (STEMI) patients the prevalence is 2%, but this included patients who were admitted for both pharmacological and mechanical reperfusion [4]. There are few limitations in identifying patients with TCM in patients who had ST elevation and underwent pharmacological reperfusion therapy. These patients would have had their coronary angiogram few days after reperfusion therapy and may have non-flow limiting lesion in their coronary arteries. This would have led to the diagnosis of TCM in few patients. The caveat to this is they may well have had thrombus in their coronary artery with no significant underlying lesion and thrombolysis would have cleared the thrombus. These patients would have been included in the TCM group leading to over-estimation of TCM in these studies. With the introduction of primary percutaneous coronary intervention (PCI) for STEMI patients, coronary angiography is performed immediately after the diagnosis of STEMI is made on the ECG and this will identify patients with coronary thrombus immediately and avoid over diagnosing TCM. 

In those patients who were included in the large multi-centre randomised Harmonizing Outcomes with Revascularization and Stents in Acute Myocardial Infarction (HORIZONS-AMI) study comparing bivalirudin with abciximab and heparin in STEMI, the prevalence of TCM was reported as 0.45% [6]. However these are selected patients and do not typically represent the characteristics of patients admitted for primary PCI in STEMI in real-world practice. So far, there is no published observational real-world data about the prevalence of TCM in patients admitted for primary PCI for STEMI. In our unit (Essex cardiothoracic centre, United Kingdom), typical TCM was noted in 17 of the 1875 patients (0.9%) admitted for primary PCI over a period of 26 months. All the 17 patients were female, giving a prevalence of 3.1% (17/560) in female patients admitted for primary PCI in our unit. The mean age of patients in our study was 70±10.7 years ranging from 56 to 94 years. The low prevalence of this condition in men may be explained by the fact that men who do develop the syndrome are more likely to die suddenly and thus do not survive till diagnosis. The other possibility is TCM itself is an exclusive female condition due to hormonal factors and the previous studies may have incorrectly diagnosed TCM in men due to the reasons discussed in the previous paragraph. 

## PATHOPHYSIOLOGY

The exact mechanism of TCM is unknown. The trigger is frequently, but not always, an intense emotional or physical stress eg, catastrophic news, death of a relative, particularly if unexpected, arguments, natural disasters (including Tsunamis), war, or even surgery. The pathogenesis is not well understood but a number of theories exist:

### Catecholamine Drive

Catecholamine activation of alpha-adrenoceptors and beta-adrenoceptors is the primary trigger of TCM changes. Catecholamine concentrates such as epinephrine and norepinephrine levels have been noted to be high during the acute phase of TCM [7]. Wittstein found that the catecholamine levels were two to three times higher in TCM patients compared to those with acute MI [7]. Abraham *et al* have reported the development of all variants of TCM in nine patients following intravenous epinephrine and dobutamine infusion, supporting the concept that catecholamines have a role in the genesis of TCM [8]. Rona recognised the role of catecholamine oxidation products in producing myocardial injury [9]. Later studies demonstrated the importance of microcirculatory effects as well as, in the norepinephrine model that of early sarcolemmal membrane permeability alteration, especially calcium ion overload. Excessive amounts of catecholamines released from sympathetic nerve endings as well as from the adrenal medulla under stressful conditions are considered to produce intracellular calcium overload and cardiac dysfunction through the β(1)-adrenoceptor signal transduction pathway. Proposed mechanisms for catecholamine-mediated stunning in TCM include epicardial vasospasm, microvascular dysfunction, hyperdynamic contractility with mid-ventricular or outflow tract obstruction, and direct effects of catecholamines on cardiomyocytes. Increase beta-2-adrenoceptor activity in the setting of a high catecholaminergic state has been proposed as possible reproducible model for this entity, inducing cardiac dysfunction and myocyte injury though calcium leakage due to hyperphosphorylation of the ryanodine receptor [10]. Studies show evidence of significant genetic influences on individual responses to adrenergic stimulation [4]. Predominant apical involvement has been explained by a denser concentration of adrenoceptors in the apex in canine heart experiments. Mori *et al. *also noted an increased beta-2 concentration gradient from apex to base commonly found in TCM [11]. These observations support the hypothesis that, during times of stress when epinephrine is the main circulating catecholamine, regional differences in adrenaline sensitive b2-receptors could explain the myocardial response to the catecholamine surge seen in TCM [12].

### Oxidative Stress

Oxidative stress can lead to myocardial necrosis, remodelling, and contractility disturbances [13-15]. The univalent reduction of oxygen (REDOX) generates reactive intermediates, such as reactive oxygen species (ROS) which can result in oxygen toxicity [13, 14]. ROS participate in the development of pathology by altering the redox state of regulatory proteins. There is now good evidence that reactive oxygen species regulate the function of calcium channels [15]. Abnormalities in calcium homeostasis underlie cardiac arrhythmia, contractile dysfunction and cardiac remodelling. The intimate link between TNF-alpha, reactive oxygen species (ROS), and mitochondrial DNA damage might also play an important role in myocardial remodelling and heart failure [16]. 

Bolli proposed three main mechanisms for myocardial stunning: 1) generation of oxygen radicals, 2) calcium overload, and 3) excitation-contraction uncoupling due to inadequate release of calcium by the sarcoplasmic reticulum [17]. The three hypotheses outlined above are not mutually exclusive and in fact may represent different steps of the same pathophysiological cascade. Thus, generation of oxyradicals (eg superoxide anion radical (O _2_.-) hydrogen peroxide (H _2 _O _2_) and hydroxyl radical (OH)) may cause sarcoplasmic reticulum dysfunction, and these processes may lead to calcium overload, which in turn could exacerbate the damage initiated by oxygen species. ROS generation can also alter the function of cardiac sodium channels, potassium channels, and ion exchangers, such as the Na/Ca exchanger [18].

There is now evidence to suggest that LV dysfunction in TCM may be related to oxidative stress in response to excess catecholamine in animal model [19]. It was demonstrated that upregulation of haemoxygenase-1 (HO-1) in cardiac and aortic macrophages using real-time reverse transcriptase polymerase chain reaction (PCR) and in situ hybridisation histochemistry and immunohistochemistry in immobilisation stressed rats [19]. HO-1 is an oxidative stress related factor, which may have a role in protecting against damage caused by reactive oxygen species. Thus, the concentration of HO-1 increases in response to increasing oxidative stress. Blocking of α- and β-adrenoceptors attenuated stress induced upregulation of HO-1 mRNA in the heart, as well as significantly altered gene expression thus favouring cardioprotection. This underlines the multiple actions exerted by adrenergic antagonists and their potentially therapeutic effects in treating TCM.

### Oestrogen Deficiency

About 90% of patients presenting with TCM are postmenopausal women. Animal models have demonstrated the cardioprotective properties of oestrogen. Ueyama *et al. *showed that when ovariectomised rats without oestradiol supplementation were exposed to immobilisation stress they demonstrated significantly greater increases in the heart rate and reduction in LV function in comparison to rats that had oestradiol supplementation [20]. Further studies on animal models are suggestive of hypothalamic adrenal downregulation by oestrogens, as well as increasing the production of cardioprotective substances like atrial natriuretic peptide and shock-protein-70 [21]. This evidence leads to the concept that postmenopausal women lose the protective effect of oestrogens, which may render them at risk of exaggerated response to circulating catecholamines.

### Transient Coronary Artery Spasm

Transient coronary artery spasm leading to transient myocardial stunning without long lasting myocardial damage was proposed as aetiology of TCM by Sato *et al*. in 1990 [1]. Some investigators reported prolonged spasm in multiple coronary arteries in patients with TCM, induced by both hyperventilation and provocative tests [22, 23]. However, only 28% of the TCM patients developed multivessel spasm on provocative tests [4], and the fact that the regional hypo/akinesis of the LV involves more than one coronary artery territory, mitigates against this possibility.

### Genetic Predisposition

Sharkey *et al*. investigated functional polymorphisms of β1 and α2c adrenergic receptors, but found no significant differences in polymorphism frequencies between TCM patients and controls [24]. It has been suggested the predominance of TCM in female subjects could be explained by their possible possession of a mutation responsible for fragile X- syndrome and cardiovascular disease, called FMR1 [25]. 

### Infective Agents

Viral illness has been considered in particular because of the infiltration by mononuclear lymphocytes and macrophages observed in histological examination of most cases. However, so far no viral agents were isolated from patients with TCM.

## CLINICAL PRESENTATION

The clinical features of TCM are important to recognize as they mimic those of acute coronary syndrome (ACS) in the absence of significant coronary artery disease. Symptoms include acute chest pain (70%) and dyspnoea (20%) [4, 26] accompanied by electrocardiographic changes, such as ST-segment elevation (30-50%) and T-wave inversions, minimal elevation of cardiac enzyme levels and transient wall-motion abnormalities. Signs and symptoms of heart failure are also common. Conversely a small proportion of patients can also be asymptomatic and only identified after presenting with abnormal ECGs, elevated biomarkers or echocardiographic features. Cardiogenic shock and ventricular arrhythmias leading to cardiac arrest are also presenting features of TCM. In our study, two out of 17 (11.8%) had out-of-hospital cardiac arrest prior to presentation to our unit, both of whom were successfully resuscitated by the paramedics. One patient was found in cardiogenic shock and had intra-aortic balloon pump (IABP) on arrival.

The Mayo Clinic published diagnostic criteria for TCM (2008) is given below: [27]

1. Transient hypokinesis, akinesis, or dyskinesis of the left ventricular mid segments with or without apical involvement; the regional wall motion abnormalities extend beyond a single epicardial vascular distribution; a stressful trigger is often, but not always, present
2. Absence of obstructive coronary artery disease or angiographic evidence of acute plaque rupture
3. New ECG abnormalities (either ST elevation and/or T wave inversion) or modest elevation in cardiac troponin
4. Absence of other precipitants eg phaeochromocytoma, myocarditis

## INVESTIGATIONS

### ECG

Electrocardiographic abnormalities are the most common finding in TCM. ST segment elevation was present in up to 56% of patients particularly in anterior leads, 39% had T wave inversion and the remaining had QT prolongation or pathological Q waves [28]. Arrhythmias such as VT, VF and Torsade de pointes have also been reported. Resolution of ECG abnormalities is frequently seen after a couple of months. 

### Cardiac Biomarkers

Cardiac enzymes such as CK and Troponin assays are typically only mildly elevated. This is disproportionate to the extent of regional wall motion abnormalities noted on imaging tests such as echocardiogram, left ventriculogram or cardiac MRI. The levels of brain natriuretic peptide (BNP), however, tend to be higher in TCM compared with acute coronary syndrome.

### Echocardiography

Echocardiography usually shows characteristic apical wall changes including hypokinesia, akinesia and dyskinesia. The overall systolic function is reduced, with the reported ejection fraction ranging from 20 to 49%. However, more recently, variations of TCM have been reported such as inverted (23%), and mid cavity (10%) wall motion abnormalities [5]. 

#### LVOT obstruction

Left ventricular outflow tract (LVOT) obstruction can occur in patients with TCM [29] and in one series there was a 25% incidence in patients diagnosed with TCM [30]. The detection of LVOT obstruction is important because they usually present with hypotension and the use of ionotropic agents may increase the intraventricular pressure gradient and induce cardiogenic shock. Vasodilators, such as nitrates, may theoretically worsen the LVOT and therefore should be avoided.

#### Right Ventricular Involvement

There have also been reports of right ventricular dysfunction in TCM. In a series by Haghi *et al*. in 2006, the RV involvement was noted in 26% of the patients with TCM.[3] The most frequently affected RV segments were apico-lateral (89%), antero-lateral (67%) and inferior segments (67%). Bilateral pleural effusions are commonly seen in patients with RV involvement.

### Cardiac Catheterisation

Almost all have unobstructed coronary arteries but a substantial proportion have incidental findings of coronary artery disease. This could merely be an innocent finding reflecting the general population. Left ventriculogram is very useful to identify TCM in acute situation when the coronary arteries are normal with ST elevation on ECG. The typical left ventriculogram in diastole (Panel A) and systole (Panel B) of TCM is shown in Fig. (**2**). 

### Cardiac Magnetic Resonance (CMR) Imaging

CMR can be extremely useful in helping differentiate TCM from different types of cardiomyopathy as well as myocarditis. Early CMR is crucial as most the imaging findings are usually present in the first 24-48 hrs followed by a complete recovery within days. Late gadolinium enhancement (LGE) is usually absent, in contrast to other causes of myocardial damage such as myocardial infarction or severe LV dysfunction due to myocarditis. Apical myocardial oedema is commonly seen in TCM on CMR correlating with LV systolic dysfunction. On T2 weighted imaging of CMR, ventricular oedema appears as high intensity signal with a diffuse or transmural distribution in this condition. Moreover, the location of the oedema is not related to a vascular territory of coronary arteries, and oedema is distributed in both the apical and mid planes of the LV, which is also helpful to differentiate from acute MI [31]. CMR can also help enable identification of thrombus in the ventricles not seen on echo. Hence, CMR can be crucial in making the correct diagnosis and should be performed on all cases of suspected TCM where possible.

### Nuclear (Iodine ^123^metaiodobenzylguanidine) Scintigraphy

^123^I-MIBG scintigraphy has been used to evaluate Takotsubo cardiomyopathy. There is decreased uptake of ^123^I-MIBG in areas of myocardial stunning. Villarroel *et al.* demonstrated a case of typical TCM and performed ^123^I-MIBG for the patient [32]. These were performed 10 days after the acute onset of symptom. This showed typical decreased uptake of ^123^I-MIBG in the distal anterior and inferior wall and apex, corresponding to the akinetic segments on left ventriculography. Rest ^99m^Tc-sestamibi images performed the following day for comparison show normal uptake in areas of decreased ^123^I-MIBG, therefore ^123^I-MIBG can be seen, notably from the inferior wall and apex [32]. These are typical features of TCM on nuclear scanning.

## MANAGEMENT

The most common presentation is chest pain with ST segment elevation in anterior leads mimicking anterior STEMI. Therefore the initial treatment is most likely to be based on suspicion of anterior STEMI. In places where there is no primary angioplasty facility, there is a risk of these patients getting thrombolysed. Therefore initial history such as emotional stress is important and TCM should be suspected in all post-menopausal women who presents with anterior STEMI. The advantage of primary angioplasty is acute STEMI can be ruled out with angiography and the left ventriculogram may clinch the diagnosis of TCM at an early stage. 

The main stay of management in patients with TCM is supportive. There is no consensus on pharmacological management of TCM and due to the rarity of this condition; no RCTs have been conducted so far. Conservative treatment frequently leads to rapid resolution. However, in the initial stages, patients are treated with Aspirin and other anti-platelet agents, their role of which will be difficult to interpret. 

The use of b-blockers has been specifically advocated due to the possible abnormal response to excessive catecholamines [27]. This is also supported by evidence from animal model studies which demonstrated that the resolution of ST segment elevation was successfully achieved by combined α and β adrenoceptor blockade [33, 34]. Furthermore, Uchida *et al*. in 2009 demonstrated that α and β adrenoceptor blockers may have a role in prevention of stress induced cardiac dysfunction [35]. Sharkey *et al.*, in 2010, however reported in their series from a single institution that 20% of patients were already on beta-blockers while they developed TCM, either first or recurrent episode [36]. They argue therefore that beta-blockers in traditional dosage did not absolutely prevent either the first or recurrent episodes of TCM.

## PROGNOSIS

The LV function starts to recover from few days and recovers completely in 3-4 weeks. Though TCM is not benign during the acute episode, there is an excellent survival outcome if managed appropriately during the acute phase. The in-hospital mortality rate varies from 1.1% to 2%. [4] In our series, over a follow up period of 22±7 months, there was no mortality. During a four year follow up, the recurrence rate was 11.4% in one series [37].

## SUMMARY

TCM is a reversible cardiomyopathy with a generally favourable outcome. It is a relatively recently described phenomenon which needs to be considered early in any patient that presents with acute dyspnoea, chest pain or collapse after an acute episode of grief, shock or stress. Multiple mechanisms potentially contribute to the pathogenesis of myocardial stunning but the exact mechanism is still not known. Catecholaminergic storm and β-adrenergic receptor-stimulated apoptosis in cardiac myocytes, mediated by reactive oxygen species/kinase-dependent activation of the mitochondrial pathway are two of the main theories. Irrespective of the cause, patients with the classic stress-induced cardiomyopathy morphology deserve special attention because this extensive distribution of wall motion abnormalities has implications for potential associated complications. CMR can be crucial in differentiating from acute MI. The management of TCM is usually conservative. The high prevalence of TCM in post-menopausal women suggests a role for oestrogen therapy. This review should provide not only a conceptual framework for further investigation of the pathophysiology of reversible cardiomyopathy but also a rationale for developing clinically applicable interventions. There is a need for national or international registry for TCM patients to understand more about this condition and manage appropriately.

## Figures and Tables

**Fig. (2):**
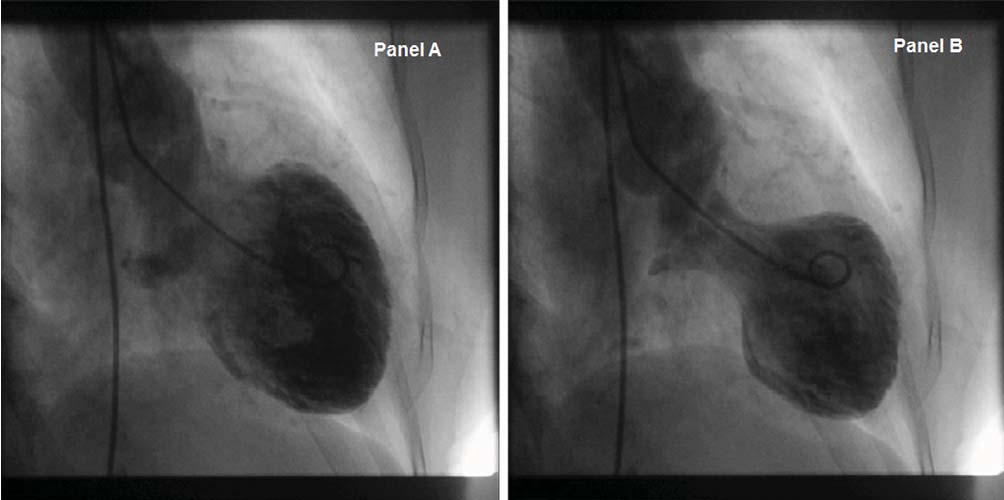
Left ventriculogram showing typical appearance of Takotsubo Cardiomyopathy in diastole (Panel A) and systole (Panel B).
